# Characterization of Native Sicilian Wines by Phenolic Contents, Antioxidant Activity and Chemometrics

**DOI:** 10.3390/molecules30030534

**Published:** 2025-01-24

**Authors:** Mattia Rapa, Martina Di Fabio, Maurizio Boccacci Mariani, Vanessa Giannetti

**Affiliations:** Department of Management, Sapienza University of Rome, Via del Castro Laurenziano 9, 00161 Rome, Italy; martina.difabio@uniroma1.it (M.D.F.); maurizio.boccaccimariani@uniroma1.it (M.B.M.); vanessa.giannetti@uniroma1.it (V.G.)

**Keywords:** phenolic compounds, Sicily, wine, antioxidant activity

## Abstract

Sicily, an island rich in history and tradition, is renowned for its unique viticultural landscape, where native vines have been cultivated for centuries. The commercial value of Sicilian wines is rooted in their distinctive flavors and aromas and the cultural heritage they embody. This paper delves into the characterization of native Sicilian wines according to their phenolic contents, antioxidant activity, and chemometrics. Nero d’Avola and Syrah showed the highest phenolic content and the highest antioxidant activity. Among the white wines, the Catarratto and Zibbibo samples were richer in antioxidants than the Grillo ones. In the Principal Component Analysis, it was possible to note the grouping of the red and white wines in the first component and an early grouping according to variety in the second one. Furthermore, the variable examined allowed for a suitable classification model (up to 83%) to be built for the Nero d’Avola, Syrah, Grillo, and Zibibbo wines through a Linear Discriminant Analysis. The findings highlight how these phenolic profiles contribute to the distinctiveness and marketability of Sicilian wines, offering a deeper appreciation of their value within the global wine industry.

## 1. Introduction

Sicily, an Italian region characterized by its rugged landscapes and rich cultural history, has long demonstrated remarkable resilience to environmental and economic challenges. This resilience is particularly evident in its agricultural practices, where local communities have adapted to the island’s diverse and often harsh conditions to cultivate unique, high-quality products. Sicilian wines stand out as a testament to the island’s agricultural ingenuity and as a symbol of its enduring connection to tradition and innovation [1,2,3,4,5,6].

The valorization of Sicilian products, particularly wine, is crucial in preserving the island’s rich agricultural heritage while boosting its economic growth. By emphasizing the unique qualities of its native vines, Sicily has successfully positioned its wines as premium products in local and international markets. This strategic focus on enhancing the value of indigenous products celebrates the region’s cultural identity. It drives sustainable development, ensuring that traditional practices are maintained and respected in the face of modern economic pressures [7,8,9,10,11,12,13].

The commercial value of Sicilian wines has seen a significant rise, fueled by the island’s commitment to quality, tradition, and innovation. Sicily’s diverse climate and fertile soils contribute to producing wines with distinctive flavors, which have garnered attention and acclaim in global markets. The island’s native grape varieties, such as Nero d’Avola and Grillo, have become highly sought after, enhancing the prestige and marketability of Sicilian wines. This commercial success boosts the local economy and reinforces Sicily’s reputation as a leading wine-producing region, where authenticity and excellence are key value drivers [14,15].

ISTAT (the Italian National Institute of Statistics) reported that Sicily’s wine production reached 6.2 million hectoliters in 2021, reflecting a 6% increase from 2020 and surpassing the ten-year average by more than 20%.

The trends in the region’s wine industry are clear: a consistent recovery following sharp declines in the recent past, a move away from bulk wines towards higher quality varieties (with PDO wines leading until 2020, although PGI wines are now seeing more notable growth), and a gradual shift in preference from red wines to white wines [16,17,18].

The enhancement of the Sicilian wine market is also due to the high differentiation of the products. Sicily boasts a remarkable array of native wines, reflecting the island’s diverse terroir and rich winemaking tradition. Among the most notable, Nero d’Avola, often referred to as the “King of Sicilian wines”, is a robust red wine known for its deep color and rich, fruity flavors with hints of plum, cherry, and spice. It offers a perfect balance of acidity and tannins, making it versatile for pairing with various dishes. Another important red wine is Syrah. Though originally from the Rhône Valley, Syrah has found a second home in Sicily, thriving in the warm climate there. Sicilian Syrah wines are bold and full-bodied, characterized by dark fruit flavors like blackberry and blackcurrant, and peppery and smoky notes. Grillo, Catarratto, and Zibibbo are the most popular for white wine. Grillo is a white grape variety central to Sicilian winemaking, producing wines with crisp acidity and fresh citrus flavors, often accompanied by notes of tropical fruit and herbs. It is particularly well suited to the island’s coastal regions, where it retains a refreshing minerality. Catarratto, instead, one of Sicily’s most widely planted white grape varieties, is known for producing wines with a light to medium body, marked by citrus and floral aromas. It is often used in blends but also shines as a varietal wine with its zesty acidity and subtle almond finish. Zibibbo is a unique grape that produces aromatic wines with intense floral and fruity notes, including hints of orange blossom, apricot, and honey. It is often used to make dry and sweet wines, including the famous Passito di Pantelleria, a dessert wine with concentrated sweetness and complex flavors. Each of these native Sicilian wines offers a distinct expression of the island’s rich viticultural heritage, showcasing the diversity and quality that have made Sicily a revered name in the world of wine [19,20,21,22].

This paper aims to comprehensively analyze the phenolic compounds in Sicilian wines, focusing on the characterization of native grape varieties through their antioxidant profiles. The study seeks to (i) identify and quantify the key phenolic compounds present in wines made from Sicilian native grape varieties, such as Nero d’Avola, Syrah, Grillo, Catarratto, and Zibibbo; (ii) assess the antioxidant activity of these wines, examining how these compounds contribute to the wine’s preservation, flavor stability, and potential health benefits; (iii) emphasize the distinctiveness of Sicilian wines by exploring how the island’s unique terroir influences the phenolic profiles and antioxidant levels of these native varieties through chemometric tools. By achieving these aims, this paper intends to enhance the understanding of the complex interplay between phenolic compounds and wine quality while reinforcing the significance of Sicily’s native vines in the global wine market.

Antioxidants are crucial in wine quality, longevity, and health benefits. These compounds, primarily phenolic, help preserve the wine by preventing oxidation, which can lead to the deterioration of flavor, color, and aroma. By protecting the wine from oxidative damage, antioxidants maintain its desired characteristics over time.

Moreover, antioxidants such as phenolic acids and resveratrol contribute to the health benefits associated with moderate wine consumption. These compounds have been linked to cardiovascular health, anti-inflammatory effects, and a reduction in oxidative stress in the body. In Sicilian wines, particularly those made from native grape varieties, the presence of these antioxidants is significant, as it not only enhances the wine’s sensory qualities but also adds to its appeal as a health-conscious choice for consumers. The high levels of antioxidants found in Sicilian wines are often attributed to the island’s unique climate and soil, which encourage the development of these beneficial compounds in the grapes.

In the literature, the determination of these substances is carried out using reversed-phase high-performance liquid chromatography (HPLC) coupled with UV–Vis spectrophotometry (HPLC-UV-Vis/HPLC-DAD) [23,24,25,26,27]. Their antioxidant activity can be studied through several in vitro methods: 1,1-diphenyl-2-picrylhydrazyl (DPPH) radical scavenging assay, ferric reducing/antioxidant power (FRAP) and Cupric Ion Reducing Capacity in the presence of neocuprine (CUPRAC); they were correlated with the Total Phenolic Content (TPC) determined with Folin–Ciocalteu’s method [26,27,28,29].

Some studies have confirmed that Sicilian wines, especially red ones such as Nero d’Avola or Syrah, have a total polyphenol content that can exceed 3500 mg/L [30,31].

Phenolic acids, including gallic acid, p-hydroxybenzoic acid, and ferulic acid, are key components of Sicilian wines and contribute significantly to their antioxidant properties [30,31,32,33,34]. Gallic acid is quantitatively one of the predominant phenols in wines. It plays a fundamental role as a precursor in the formation of hydrolysable tannins and is trapped within the condensed tannins [35].

In general, red wines contain more resveratrol than white wines, as resveratrol is mainly found in the skin of grapes. In Sicily, some wines, such as Nero d’Avola, may contain significant levels of resveratrol: the average levels of trans-resveratrol in red wines range from 0.5 to 15 mg/L, while in white wines, the concentration of trans-resveratrol is generally between 0.1 and 1 mg/L [31,32,33].

For these reasons, this paper has determined that gallic acid, p-hydroxybenzoic acid, ferulic acid, and t-resveratrol are the most representative phenolic compounds in this matrix, along with their antioxidant activity, in Sicilian wines.

To the best of our knowledge, this is the first study performing this kind of characterization for Grillo, Zibibbo, and Catarattatto wines. Furthermore, for Nero d’Avola and Syrah, this type of integrated characterization by means of four phenolic compounds and TPC, DPPH, FRAP, and CUPRAC antioxidant evaluation has never been reported in the literature. Therefore, this study could be used by stakeholders of the supply chain and as a benchmark for future studies.

## 2. Results and Discussion

### 2.1. Analytical Results

The analyses compare various antioxidant and phenolic content measures in different grape varieties: Catarratto, Grillo, Nero d’Avola, Syrah, and Zibibbo.

The results cover the Total Phenolic Content (TPC), DPPH radical scavenging activity, Ferric Reducing Antioxidant Power (FRAP), Cupric Ion Reducing Antioxidant Capacity (CUPRAC), and specific phenolic compounds such as gallic acid, p-hydroxybenzoic acid, ferulic acid, and trans-resveratrol (Table 1).

**Figure 1:**
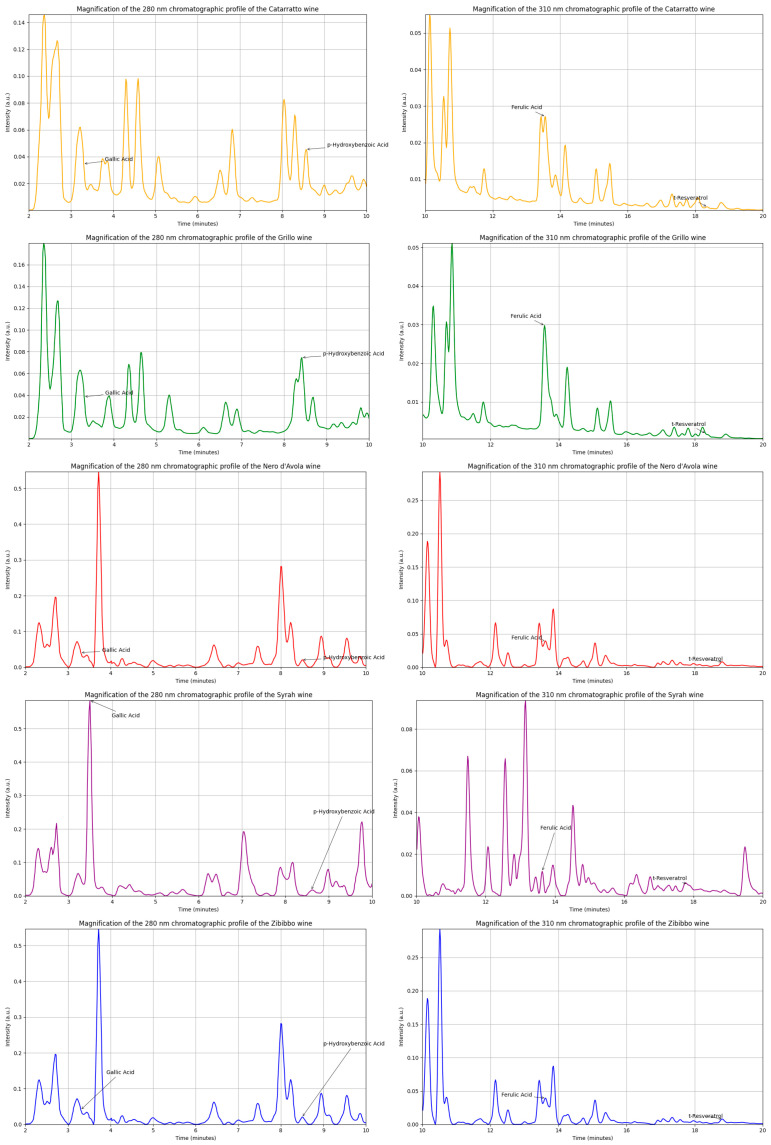
Chromatographic profiles of four phenolic compounds determined in Cataratto (yellow), Grillo (green), Nero D’Avola (red), Syrah (purple), and Zibibbo (blue).

The chromatographic results of gallic acid, p-hydroxybenzoic acid, ferulic acid, and t-resveratrol are reported in Figure 1.

The gallic acid content is substantially higher in Nero d’Avola (152 mg/L) and Syrah (144 mg/L) than the other varieties, which fall below 16 mg/L. Gallic acid is known for its potent antioxidant properties, contributing to the higher antioxidant measures observed in these two grape varieties. p-hydroxybenzoic acid, ferulic acid, and t-resevratrol follow a similar pattern, with Syrah and Nero d’Avola showing much higher concentrations than the others, contributing to the antioxidant profile of these grape varieties.

Nero d’Avola and Syrah also show a significantly higher TPC (1490 mg/L GAE and 1380 mg/L GAE, respectively) than the other grape varieties, indicating a richer content of phenolic compounds known for their antioxidant properties. Catarratto, Grillo, and Zibibbo (245 mg/L GAE) have much lower TPC levels, suggesting that they possess fewer total phenolic compounds.

A similar trend was observed for the antioxidant capacity assays. The red wines exhibit the highest antioxidant activity in DPPH (Nero d’Avola: 5370 μM TAEC, Syrah: 5280 μM TAEC), FRAP (Nero d’Avola: 14,600 μM TAEC, Syrah: 11,000 μM TAEC), and CUPRAC (Syrah: 13,200 μM TAEC, Nero d’Avola: 13,000 μM TAEC).

The data clearly indicate that Nero d’Avola and Syrah exhibit a superior antioxidant activity and phenolic content compared to those of Catarratto, Grillo, and Zibibbo. This can be attributed to the higher levels of total phenolics, including specific compounds like gallic acid and resveratrol, potent antioxidants. Due to their relatively lower phenolic contents, Catarratto, Grillo, and Zibibbo may have lower antioxidant capacities.

These differences can be attributed to several factors.

Phenolic compounds, including tannins and flavonoids, are concentrated in the skins and seeds. During winemaking, grapes undergo maceration, leaving the juice in contact with the skins, leading to the greater extraction of phenolic compounds. Red wines typically undergo longer maceration and fermentation periods, allowing for the more thorough extraction of phenolics from the grape skins. In contrast, white wines often have minimal skin contact, resulting in a lower phenolic content.

Additionally, aging in oak barrels or other vessels can influence the phenolic content through oxidation and interaction with wood components. Thus, winemaking choices can enhance or diminish the phenolic profiles of different grape varieties.

These findings suggest that Nero d’Avola and Syrah grapes may be more beneficial for health-related applications due to their stronger antioxidant properties. These grapes could be helpful in wine production or as natural sources of antioxidants.

To the best of our knowledge, this is the first paper to analyze Catarratto, Grillo, and Zibibbo white wines. Nevertheless, it is possible to compare the results of this study with those of other white wines produced in Italy and worldwide. Gallic acid was found in a range of 0.02–31 mg/L, p-hydroxybenzoic acid at around 0.1–38 mg/L, ferulic acid at around 0.05–5.5 mg/L, and t-resveratrol at around 0.1–1.61 mg/L [36,37,38,39,40]. These values agree with our results, as reported in Table 1.

Furthermore, for red wines, the results could be compared with those of other studies in the literature on the same Sicilian wines.

For Nero D’Avola, a comparison of our data with the literature was made in Table 2. The TPC value of the present study appears lower than that of the literature studies. The current study provides more comprehensive antioxidant activity metrics using multiple assays (DPPH, FRAP, and CUPRAC), which are unavailable in older studies, limiting direct comparisons. For the single phenolic compounds, the current study measured a higher value of gallic acid (152 ± 27 mg/L vs. 28.34–100.73 mg/L) and ferulic acids (7.03 ± 4.42 mg/L vs. 0.13–1.96 mg/L). The p-hydroxybenzoic acid was detected only in the current study, while the t-resveratrol level is similar to the range reported in other studies.

It is noteworthy that the production year of the examined wine is different. The wine analyzed in this study comes from the 2019–2021 campaign, while those found in the literature come from the 2002–2004 campaign.

While the total phenolics have decreased, compounds like gallic acid, ferulic acid, and p-hydroxybenzoic acid show relatively higher concentrations in the newer samples.

Table 3 reports a similar comparison for Syrah wine. In this case, as with another study on Sicilian Syrah, results from Syrah wines from other countries (EU and non-EU) have also been reported.

The TPC values of the Syrah wines in this study were lower than those found in other Sicilian wines, but are slightly higher than those of Portuguese wines. For the antioxidant activity, it was possible to compare the DPPH results. The current study records antioxidant activity at 5280 ± 417 μM TAEC. At the same time, wines from Macedonia ranged from 3340 to 4010 μM TAEC, and wines from Brazil, Chile, Australia, and South Africa Dantas have a lower range (1301–2099 μM TAEC). The higher antioxidant values suggest that the Sicilian Syrah wines display strong antioxidant activity, even if their TPC value is relatively lower.

For the single phenolic compounds, the gallic acid level in the current study (144 ± 29 mg/L) is higher than that reported in other Sicilian wines (39.07–106.66 mg/L) and Portuguese wines (20.73–25.89 mg/L). A similar trend was found for ferulic acid, which was at a higher concentration in this study (5.80 ± 4.16 mg/L) compared to the lower levels in the other Sicilian wines (0.04–1.34 mg/L) and Portuguese wines (0.10–0.15 mg/L). p-hydroxybenzoic acid was detected in the current study at 86.3 ± 18.5 mg/L, a level much higher than that in wines from Portugal (0.19–0.24 mg/L). The current study reports 0.529 ± 0.334 mg/L of t-resveratrol, which is within the range reported by the other Sicilian studies (0.10–0.88 mg/L) and comparable to wines from different regions like Brazil and Chile (0.59–2.06 mg/L). The higher concentrations of specific phenolic compounds in this study, such as gallic acid and ferulic acid, indicate variability in the phenolic profile that may enhance certain aspects of antioxidant activity.

### 2.2. Chemometric Analysis

The analytical results of the single phenolic compounds and antioxidant activity determination have highlighted specific markers for wine typologies (red/white) and some particular wine typologies. Various chemometrics tools have been applied to the data matrix to enhance characterization and create classification models to differentiate the wine samples. The first chemometric application was a Principal Component Analysis (PCA).

**Figure 2:**
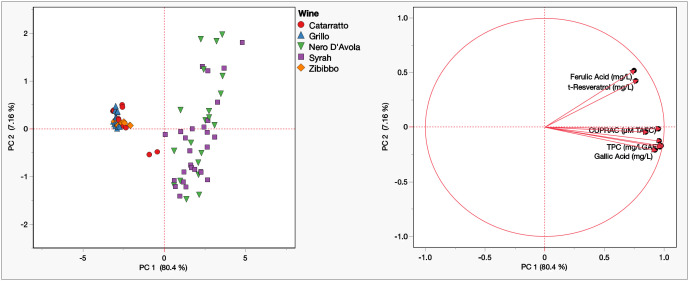
Scores and loading plot from PCA on Sicilian wines: analytical results.

A PCA was performed to highlight the natural grouping of samples. The data set underwent an autoscaling pretreatment to exclude the variance related to the different measurement units. The scores and loading plot of the unsupervised PCA are reported in Figure 2.

The first two PCs explained 87.5% of the total variance. This can be highlighted as an early grouping of the samples. The Nero D’Avola and Syrah samples (red wines) appeared in the right part of the score plot, while the Catarratto, Grillo, and Zibibbo samples (all the white wines) were all located in the left part. Applying the Kaiser criterion and scree graph evaluation, the first two PCs were chosen as significant. From the score plot, it is also possible to highlight that the separation of the red/white wines is due to the first PC, while an initial internal separation between the various types of wine appeared in the second PC.

Table 4 reports the percentage contribution of the variables in each PC. Notably, all the variables analyzed contribute almost equally to the construction of the first PC and are, therefore, all necessary to distinguish the red wines from the white wines. On the other hand, the second PC is predominantly influenced by ferulic acid (64.8%), to which the separation between the various wine samples could be attributed.

**Figure 3:**
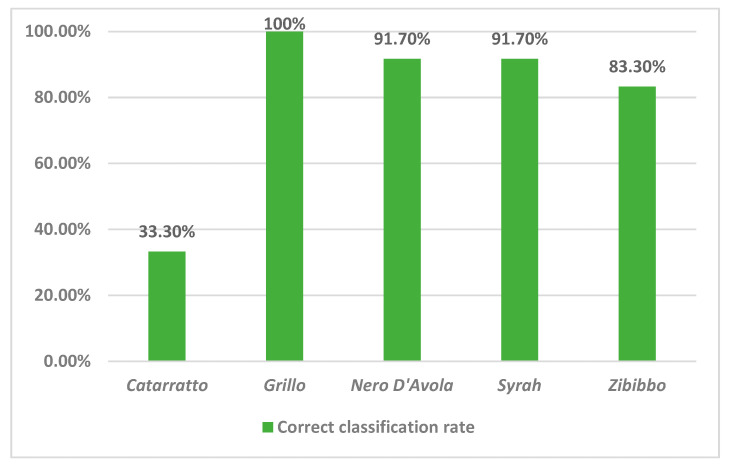
Percentage of correct classification of wine typologies by LDA.

Once the natural grouping of the samples was highlighted in the PCA, the data were used to build a recognition tool for the five types of wines analyzed. A Linear Discriminant Analysis (LDA) was tested to classify the samples according to their typology. Table 5 reports the confusion matrix of the wine samples according to their varieties, while Figure 3 reports the correct classification rates.

The values along the diagonal in the confusion matrix represent the correctly classified samples for each grape variety. Grillo has the highest number of correct classifications (12 out of 12), indicating that the classification model performs very well for this variety. Nero D’Avola and Syrah also show a strong classification performance, with 91.70% of the samples correctly classified. Zibibbo has ten correct classifications, indicating a good performance, though not perfect (83.3%). The classification model performs well for these grape varieties, suggesting they have distinct, easily identifiable characteristics.

On the other hand, Catarratto has a lower accuracy, with only 4 out of 12 samples correctly classified. Misclassifications occur when the samples are placed in a different category. For Catarratto, six samples were misclassified as Grillo and two as Zibibbo, indicating overlapping characteristics among these varieties. The high number of misclassifications suggests that the distinguishing features of Catarratto are less pronounced or that there is some overlap with other varieties, particularly Grillo and Zibibbo.

Additional distinguishing features could be considered to enhance the classification accuracy, or further refinement of the model may be necessary, especially for varieties like Catarratto.

Overall, the table indicates a generally effective classification system, with some areas that could benefit from further investigation and adjustment.

## 3. Materials and Methods

### 3.1. Reagents and Standard Solutions

All reagents used were analytical-grade. The acetonitrile, methanol, and formic acid were provided by Merck (Darmstadt, Germany). The water was obtained from a Milli-Q water purification system (Millipore, Bedford, MA, USA). The standards of gallic acid (r^2^ = 0.9981), p-hydroxybenzoic acid (r^2^ = 0.9995), ferulic acid (r^2^ = 0.9999), and t-resveratrol (r^2^ = 0.9997) were from Sigma-Aldrich (St. Louis, MO, USA). The standard stock solutions of polyphenols were prepared in methanol and stored at 4 °C in the dark. Trolox (6-hydroxy-2,5,7,8-tetramethylchromane-2-carboxylic acid), Folin–Ciocalteu reagent, 2,4,6-tris(2-pyridyl)-s-triazine (TPTZ), Copper (II) chloride, iron (III) chloride, sodium acetate, ammonium acetate, sodium carbonate, and neocuprine were purchased from Merck (Darmstadt, Germany). The standard stock solutions of Trolox were prepared in methanol and used immediately as soon as they were prepared.

### 3.2. Sampling

**Figure 4:**
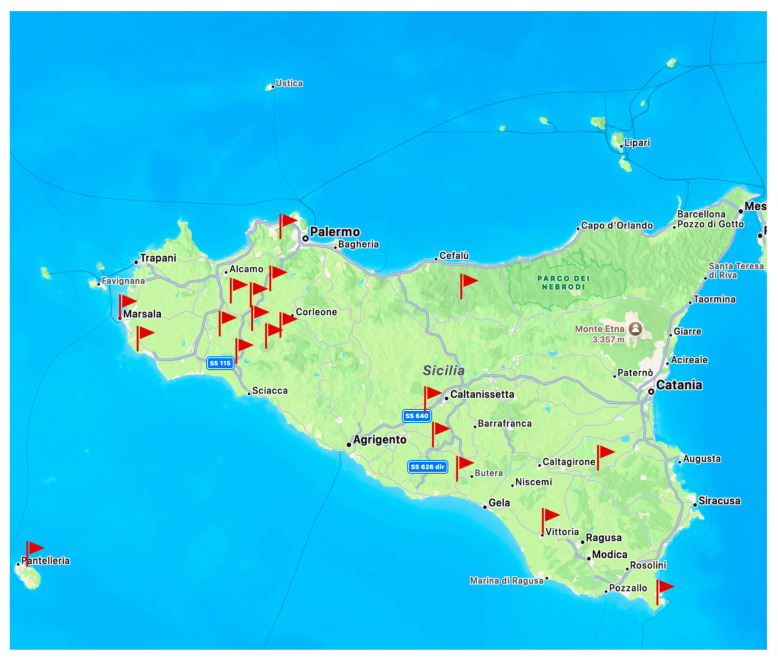
Location of the wineries sampled in this study.

A total of fifty wines from Sicilian wineries were analyzed. The samples are of the Grillo (*n* = 12), Catarratto (*n* = 12), Zibibbo (*n* = 12), Nero D’Avola (*n* = 12), and Syrah (*n* = 12) varieties. The wineries are located in the following Sicilian municipalities: Grisi, Marzamemi, Serradifalco, Riesi, Partinico, Menfi, Butera, Santa Ninfa, Petrosino, Camporeale, Pantelleria, Sambuca di Sicilia, Contessa Entellina, Vittoria, Castelbuono, Licodia Eubea, Marsala, Salaparuta, and Nuova Gibellina, located in the provinces of Agrigento, Palermo, and Trapani, as reported in Figure 4. The samples were produced from 2019 to 2021. Two bottles from different production batches were collected for the same year of production.

### 3.3. HPLC Analysis

The samples were analyzed immediately after opening the bottles. Before the chromatographic analysis, the sample was filtered on a disposable acetate syringe filter of 0.45 μm cellulose [24,25]. A Waters HPLC system (600 Waters, Milford, MA, USA), which was entirely assembled with PEEK tubing, was used for the chromatographic analysis. The HPLC system was equipped with a 20 μL injection loop and coupled to a Waters Photodiode Array Detector (2998 Waters, Milford, MA, USA) set at 280 nm. A reversed-phase Kinetex C18 column (250 × 4.6 mm i.d., 5 μm pore size) (Phenomenex, Torrance, CA, USA) was used. The mobile phase consisted of a 0.5% (*v*/*v*) solution of formic acid (eluent A) and acetonitrile (eluent B). The elution gradient was optimized in a previous study [42]. The elution gradient was as follows: 90:10% (A:B) from 0 to 2 min, transition to 85:15% for 13 min, then 50:50% for 2 min, and finally, 10:90% for 12 min. This was followed by column cleaning and re-equilibration. The chromatographic run was 35 min. Data acquisition and processing were performed using the Empower v2 software. Gallic acid, p-hydroxybenzoic acid, ferulic acid, and t-resveratrol were identified by comparing their retention times with those of the pure standards, and their quantification was carried out using the external standard method at 280 nm for gallic acid and p-hydroxybenzoic acid and at 310 nm for ferulic acid and t-resveratrol.

### 3.4. Determination of Total Phenolic Content

Spectrophotometric determination of the TPC was carried out using the Folin–Ciocalteu method with some modifications [26], using gallic acid as the standard. For the preparation of the calibration curve, 50 μL aliquots of 200, 300, 400, 500, and 600 mg/L aqueous gallic acid solutions or 50 μL of diluted samples of wines (1:1, wine/water) were introduced into a test tube. Then, 1.5 mL of 2% sodium carbonate was added. After incubation for 2 min, 250 μL of Folin–Ciocalteu’s reagent (diluted with water 1:1, *v*/*v*) was added. After a further 30 min, the absorbance was measured at 750 nm using a spectrophotometer. Results are expressed as mg gallic acid equivalents per liter of wine (mg GAE/L).

### 3.5. Determination of Antioxidant Activity

#### 3.5.1. DPPH Assay

1,1-Diphenyl-2-Picrylhydrazyl Radical (DPPH∙) is a free radical that accepts an electron or hydrogen atom from donor molecules, changing its color from purple to yellow [43]. The procedure consisted of adding 100 μL of wine; for red wines, a 1:4 dilution in methanol was performed with 2.9 mL of a DPPH radical methanolic solution (0.05 mg/mL); and, after 30 min, the percentage of absorbance decrease was measured at 515 nm [42]. The percentage inhibition of the initial concentration of DPPH· radical was calculated as follows:

(1) $$\%I=\frac{A_{DPPH}-A_{wine}}{A_{DPPH}}\times 100$$

Calibration curves obtained from methanolic solutions of Trolox (5–1000 μM) quantified antioxidant capacity.

#### 3.5.2. Ferric Reducing Antioxidant Power (FRAP)

The FRAP method evaluates antioxidant activity by measuring the reduction of 2,4,6-tris(2-pyridyl)-s-triazine (TPTZ) to the ferrous form Fe (II). The FRAP assay was performed according to the method described previously [29]. The FRAP solution consisted of 25 mL of acetate buffer (300 mM, pH 3.6), 2.5 mL of a 10 mM TPTZ (2,4,6-tripyridyl-s-triazine) solution in 40 mM HCl, and 2.5 mL of 20 mM FeCl3. The mixture was freshly prepared and was heated to 37 °C prior to use. A total of 150 µL of samples (white wines diluted 1:5 in water; red wines diluted 1:40 in water) or the Trolox standard solution was combined with 2.85 mL of the FRAP solution. After 30 min, the absorbance was recorded at 593 nm.

Aqueous solutions of Trolox concentration were between 100 and 300 μM for calibration. Results are expressed as μM Trolox Equivalent Antioxidant Capacity (μM TAEC).

#### 3.5.3. Cupric Reducing Antioxidant Capacity (CUPRAC)

In the CUPRAC assay, the primary reagent is copper, which is reduced by the sample’s polyphenols and then undergoes neocuprine chelation, which accelerates the reaction by raising the reagent’s redox potential [43]. The main CUPRAC method [44] was adapted for the study of wine using Trolox as the standard. A volume of 0.8 mL each of 10 mmM Cu(II), 7.5 mM neocuprine, and 1 M potassium phosphate buffer (pH 7) solutions and 0.48 mL water were mixed in a test tube. A volume of 0.4 mL of Trolox standard solution or samples (red wines diluted 1:12 in water; white wines diluted 1:4 in water) was added to the initial mixture. The absorbance was measured at 450 nm after 30 min. Trolox standard solutions were prepared at a concentration range from 40 to 400 μM. Results are expressed as μM Trolox Equivalent Antioxidant Capacity (μM TAEC).

#### 3.5.4. Figures of Merit

Figures of merit of the methods mentioned above are reported in Table 6.

In order to assess the performance of the calibration curve in describing the relationship between measured signals and known sample concentrations, the coefficient of determination (R^2^) was determined from the calibration curve. This was achieved by considering the sum of residual squares and the total sum of squares. The linear range of the calibration curve was determined by constructing a linear regression line (y = mx + q) on the calibration data and verifying a coefficient of determination R^2^ ≥ 0.99. The lower limit of detection (LOD) is the minimum analyte concentration that can be reliably detected within the experimental conditions of the method. However, its quantitative determination is not necessarily guaranteed. The LOD is determined by the standard deviation (SD), represented by σ, of repeated measurements of a sample at low concentration and the slope, denoted by m, of the calibration curve. The LOD was calculated according to Equation (2):

(2) LOD=3.3σ/m

Precision, the degree of agreement between repeated measurements of the same sample, was calculated as the relative standard deviation (RSD%) between replicate analyses. Accuracy, defined as the closeness between the measured value and the true value, was expressed as the relative error (%RE) concerning the theoretical expected value. Both parameters were evaluated on samples at known concentrations to validate the analytical method.

#### 3.5.5. Statistical Analysis

HSD Tukey test and chemometric data analyses (PCA and LDA) were performed with JMP software (Version 17 Pro, SAS Institute, Cary, NC, USA).

## 4. Conclusions

Sicily is one of the leading Italian regions for wine production, with many native wines reflecting the island’s diverse terroir and winemaking traditions.

The comparative analysis of Sicilian grape varieties highlights significant differences in the antioxidant capacity and phenolic content across the studied wines. The red grape varieties, particularly Nero d’Avola and Syrah, demonstrated superior antioxidant activity and higher Total Phenolic Content (TPC) values and amounts of individual phenolic compounds such as gallic acid, ferulic acid, and t-resveratrol compared to the white grape varieties (Catarratto, Grillo, and Zibibbo). These differences are likely due to winemaking practices, such as extended maceration and fermentation in red wines, which facilitate greater extraction of phenolic compounds from the grape skins. These findings suggest that Sicilian red wines, especially Nero d’Avola and Syrah, may offer more significant health benefits due to their robust phenolic profiles and antioxidant properties.

The Principal Component Analysis (PCA) revealed a natural separation between the red and white wines, with the first two principal components accounting for 86.5% of the total variance. The red wines, Nero d’Avola and Syrah, were distinguished from the white wines (Catarratto, Grillo, and Zibibbo) primarily based on the first principal component. In contrast, in the second principal component, an early differentiation of grape varieties occurred, mainly influenced by ferulic acid. The Linear Discriminant Analysis (LDA) further demonstrated the model’s classification effectiveness, with high accuracy rates for Grillo, Nero d’Avola, Syrah, and Zibibbo. However, Catarratto showed a lower classification accuracy due to its overlapping characteristics with Grillo and Zibibbo.

These findings suggest that while the current classification model is generally effective, further refinement and the inclusion of additional distinguishing markers could improve the accuracy, particularly for closely related varieties like Catarratto. Overall, this study enhances the understanding of the phenolic profiles and antioxidant characteristics that differentiate Sicilian wines, providing a foundation for more precise wine classification and quality assessment.

## Figures and Tables

**Table 1:** Phenolic compound content and antioxidant activity of analyzed Sicilian wines, expressed as mean of three measurements of each sample ± standard deviation. Rows not connected by the same letter are statistically different (*p* < 0.05). TPC: Total Phenolic Content; DPPH: 1,1-Diphenyl-2-Picrylhydrazyl; FRAP: Ferric Reducing Antioxidant Power; CUPRAC: Cupric Reducing Antioxidant Capacity.

	Catarratto	Grillo	Nero D’Avola	Syrah	Zibibbo
TPC (mg/L GAE)	294 ± 313 ^b^	182 ± 22.2 ^b^	1490 ± 110 ^a^	1380 ± 155 ^a^	245 ± 93.1 ^b^
DPPH (μM TAEC)	998 ± 1470 ^b^	341 ± 89.5 ^b^	5370 ± 446 ^a^	5280 ± 417 ^a^	395 ± 167 ^b^
FRAP (μM TAEC)	2170 ± 2720 ^c^	944 ± 145 ^c^	14,600 ± 1910 ^a^	11,000 ± 1260 ^b^	1170 ± 356 ^c^
CUPRAC (μM TAEC)	1930 ± 1880 ^b^	1190 ± 300 ^b^	13,000 ± 3620 ^a^	13,200 ± 3540 ^a^	1900 ± 905 ^b^
gallic acid (mg/L)	9.44 ± 3.15 ^b^	8.17 ± 3.76 ^b^	152 ± 27 ^a^	144 ± 29 ^a^	15.4 ± 5.4 ^b^
p-hydroxybenzoic Acid (mg/L)	33 ± 22.8 ^c^	12 ± 5.18 ^d^	70.9 ± 21.2 ^b^	86.3 ± 18.5 ^a^	34.4 ± 13.9 ^c^
ferulic fcid (mg/L)	1.33 ± 0.52 ^b^	1.05 ± 0.72 ^b^	7.03 ± 4.42 ^a^	5.80 ± 4.16 ^a^	0.63 ± 0.53 ^b^
t-resveratrol (mg/L)	0.123 ± 0.034 ^b^	0.113 ± 0.022 ^b^	0.620 ± 0.385 ^a^	0.529 ± 0.334 ^a^	0.101 ± 0.014 ^b^

**Table 2:** Comparison of phenolic compound content and antioxidant activity results of our paper and literature for Nero D’Avola wines. TPC: Total Phenolic Content; DPPH: 1,1-Diphenyl-2-Picrylhydrazyl; FRAP: Ferric Reducing Antioxidant Power; CUPRAC: Cupric Reducing Antioxidant Capacity.

Nero D’Avola			
Reference	This Study	[30]	[34]
Origin	Sicily	Sicily	Sicily
Production year	2019–2021	2003–2004	2002
TPC (mg/L GAE)	1490 ± 110	2360–3730	2563–4209
DPPH (μM TAEC)	5370 ± 446	-	-
FRAP (μM TAEC)	14,600 ± 1910	-	-
CUPRAC (μM TAEC)	13,000 ± 3620	-	-
gallic acid (mg/L)	152 ± 27	-	28.34–100.73
p-hydroxybenzoic acid (mg/L)	70.9 ± 21.2	-	-
ferulic acid (mg/L)	7.03 ± 4.42	-	0.13–1.96
t-resveratrol (mg/L)	0.620 ± 0.385	-	0.12–0.62

**Table 3:** A comparison of phenolic compound content and antioxidant activity results from our paper and the literature for Syrah wines. TPC: Total Phenolic Content; DPPH: 1,1-Diphenyl-2-Picrylhydrazyl; FRAP: Ferric Reducing Antioxidant Power; CUPRAC: Cupric Reducing Antioxidant Capacity.

Syrah						
	This Study	[30]	[34]	[28]	[41]	[35]
Origin	Sicily	Sicily	Sicily	Portugal	Macedonia	Brazil, Chile, Australia, South Africa
Production year	2019–2021	2003–2004	2002	2008–2012	2012	2011–2019
TPC (mg/L GAE)	1380 ± 155	3000–3410	3110–3900	1804–1992	-	-
DPPH (μM TAEC)	5280 ± 417	-	-	-	3340–4010	1301–2099
FRAP (μM TAEC)	11,000 ± 1260	-	-	-	-	-
CUPRAC (μM TAEC)	13,200 ± 3540	-	-	-	-	-
gallic acid (mg/L)	144 ± 29	-	39.07–106.66	20.73–25.89	-	42.42–81.90
p-hydroxybenzoic acid (mg/L)	86.3 ± 18.5	-	-	0.19–0.24	-	-
ferulic acid (mg/L)	5.80 ± 4.16	-	0.04–1.34	0.10–0.15	-	-
t-resveratrol (mg/L)	0.529 ± 0.334	-	0.10–0.88	0.11–0.26	-	0.59–2.06

**Table 4:** Percentage contribution of variables in PC building. TPC: Total Phenolic Content; DPPH: 1,1-Diphenyl-2-Picrylhydrazyl; FRAP: Ferric Reducing Antioxidant Power; CUPRAC: Cupric Reducing Antioxidant Capacity.

	PC 1	PC 2
TPC (mg/L GAE)	14.4	5.3
DPPH (μM TAEC)	14.2	5.2
FRAP (μM TAEC)	14.2	2.9
CUPRAC (μM TAEC)	14.2	0.3
gallic acid (mg/L)	13.5	7.1
p-hydroxybenzoic Acid (mg/L)	10.6	0.0
ferulic acid (mg/L)	8.9	64.8
t-resveratrol (mg/L)	10.0	14.2

**Table 5:** Confusion matrix of wine samples classified by LDA.

	Catarratto	Grillo	Nero D’Avola	Syrah	Zibibbo
Catarratto	4	6	0	0	2
Grillo	0	12	0	0	0
Nero D’Avola	0	0	11	1	0
Syrah	0	0	1	11	0
Zibibbo	0	2	0	0	10

**Table 6:** Figures of merit of methodologies involved in the study.

	R^2^	Linearity Range	LOD	Precision	Accuracy
TPC	0.9978	200–600 mg/L	165 mg/L	2%	93%
DPPH	0.9953	25–1000 μM	10 μM	2%	88%
FRAP	0.9985	100–1000 μM	80 μM	1%	97%
CUPRAC	0.9977	50–500 μM	30 μM	1%	96%
Gallic Acid	0.9981	0.4–150 mg/L	0.30 mg/L	0.5%	95%
p-Hydroxybenzoic Acid	0.9995	0.4–90 mg/L	0.33 mg/L	0.5%	93%
Ferulic Acid	0.9999	0.4–100 mg/L	0.27 mg/L	0.5%	95%
t-Resveratrol	0.9997	0.1–100 mg/L	0.05 mg/L	0.5%	90%

## Data Availability

Data is contained within the article.
